# Using the mouse grimace scale and behaviour to assess pain in CBA mice following vasectomy

**DOI:** 10.1016/j.applanim.2016.05.020

**Published:** 2016-08

**Authors:** Amy L. Miller, Gemma L. Kitson, Benjamin Skalkoyannis, Paul A. Flecknell, Matthew C. Leach

**Affiliations:** a School of Agriculture, Food and Rural Development, Agriculture Building, Newcastle University, Newcastle upon Tyne, NE1 7RU, UK; b Institute of Neuroscience, Comparative Biology Centre, The Medical School, Newcastle University, Newcastle upon Tyne, NE2 4HH, UK

**Keywords:** Mouse, Vasectomy, Pain, Behaviour, Mouse grimace scale

## Abstract

Mice used in biomedical research should have pain reduced to an absolute minimum through refinement of procedures or by the provision of appropriate analgesia. Vasectomy is a common and potentially painful surgical procedure carried out on male mice to facilitate the production of genetically modified mice. The aim of our study was to determine if 0.05 mg/kg buprenorphine would ameliorate pain associated changes following abdominal vasectomy and to determine if the mouse grimace scale is an appropriate tool for the assessment of pain in this model. Eight male CBA mice underwent abdominal vasectomy as part of a genetically modified mouse-breeding programme. Here we assessed pain using a previously validated behaviour-based method and the mouse grimace scale. All mice received buprenorphine (0.05 mg/kg s.c.) pre-surgery. Behaviour and grimace scores were compared between baseline (pre-surgery), 30 min, 5 h, 24 h and 25 h post surgery. Following 24 h post-op, all mice were administered 5 mg/kg meloxicam (s.c.) as additional analgesia. Significant increases in specific pain behaviours and mouse grimace scale score were found 30 min post surgery. At 5 h post surgery, scores were returning to baseline levels. Frequency of rearing was significantly decreased at both 30 min and 5 h post surgery compared to baseline, demonstrating a longer lasting change in normal exploratory behaviour. Buprenorphine (0.05 mg/kg) was ineffective at ameliorating these pain-associated changes in CBA mice and should be considered inadequate at this dose. By 24 h post surgery, pain associated behaviours, grimace scale and rearing had all returned to baseline levels. There was no change in pain behaviours or MGS following administration of meloxicam indicating that an additional dose of meloxicam does not appear to offer benefit at this point. Using the mouse grimace scale to assess pain in mice, appeared to be effective in the immediate post vasectomy period in CBA mice demonstrating the same duration of increased score as the pain associated behaviours.

## Highlights

• MGS score significantly increases following vasectomy in CBA mice.
• Buprenorphine (0.05 mg/kg) does not prevent pain associated changes in behaviour.
• Baseline MGS scores in male CBA mice are not zero.

## 1 Introduction

Prevention or alleviation of pain in laboratory animals is a fundamental requirement of in vivo research. In 2013, 3.08 million mice were used in regulated procedures in the UK, with over 449,000 undergoing general anaesthesia with recovery (Home Office, 2014), often for the purposes of surgery. The production of GM mice requires the use of vasectomised males to induce pseudo pregnancy (Ittner and Götz, 2007) and this provides a useful model for assessing the pain associated with surgery. A number of previous studies have assessed pain following both scrotal and abdominal approach vasectomy and have identified key changes in behaviour considered to be pain related (Leach et al., 2012, Miller et al., 2012, Wright-Williams et al., 2007). Although vasectomy via the scrotal approach was predicted to be less painful (Robinson et al., 2003), data has shown that there is likely no significant advantage to one approach over the other (Miller et al., 2012). A number of other studies have used this model, with the behaviour-based scoring system, to evaluate analgesic efficacy (e.g. Wright-Williams et al., 2013).

Behaviour-based scoring is very time consuming to carry out, so novel methods of assessing pain and analgesia efficacy that take less time to implement would offer a distinct advantage. The mouse grimace scale (MGS), devised by Langford et al. (2010) shows promise in this area as it is described as accurate and reliable with scoring requiring significantly less time than full behavioural analysis. The MGS comprises five facial action units (FAUs), orbital tightening, cheek bulge, nose bulge, ear position and whisker position. These FAUs are scored separately on a 3 point scale and then combined to produce an overall ‘grimace score’.

To date this method has undergone initial validation in the assessment of scrotal approach vasectomy in CD1 mice (Leach et al., 2012), demonstrating a significant increase in MGS score following surgery that could be reduced by the administration of either 20 mg/kg (sc) meloxicam or 5 mg/kg local infusion of the scrotum of bupivacaine. This pattern was also demonstrated when manually scoring key validated pain associated behaviours, with a high positive correlation between the two methods.

Here we aimed to study a different yet common strain of laboratory mouse, CBA, to determine if the MGS may also be an effective method of pain assessment in this strain. Previous work has demonstrated that neither isoflurane anaesthesia nor subcutaneous administration of buprenorphine results in any changes in MGS score or presence of ‘pain behaviours’ in control CBA mice that have not undergone a painful procedure (Miller et al., 2015). Consequently, any changes that are demonstrated here are likely due to the presence of pain. The dose of buprenorphine selected was 0.05 mg/kg. This dose has been previously shown to be the lowest dose required to significantly reduce MGS score in CD1 mice following laparotomy (Matsumiya et al., 2012) and significantly reduce pain specific behaviours in C57Bl/6 and C3H mice (Wright-Williams et al., 2013).

## 2 Materials and methods

### 2.1 Ethical statement

All procedures were conducted in accordance with the Animals (Scientific Procedures) Act 1986, European Directive EU 2010/63 and with the approval of the Animal Welfare and Ethics Review Board at Newcastle University. All mice that were vasectomised in this study were required for use in the university’s genetically modified mouse production programme; consequently, no animals underwent surgery solely for the purpose of this study. Previous studies have utilised similar numbers of mice following appropriate power analysis (Leach et al., 2012). This study employed a strict ‘rescue’ analgesia policy. If any animal was deemed to be in greater then mild pain (assessed by an independent veterinarian), then buprenorphine (0.1 mg/kg sc) was to be immediately administered and the animal was removed from the study. No animals were deemed to require any additional analgesia. Animals acted as their own controls to remove the effect of within group variation and reduce the total number of animals used. Previous study (Wright-Williams, 2007) has indicated that vasectomy results in post-operative pain and therefore it was decided a control group with no analgesia was not appropriate in this case. A sham group was also not included as this has been carried out previously and no change in pain associated behaviours were found (Wright-Williams, 2007).

### 2.2 Animals

Eight CBA male mice (Charles River Laboratories Inc, Kent) weighing 25.6–28.7 g at the start of the study were used. Mice were housed in groups of 4 in individually ventilated cages (IVCs) (Type 2–Arrowmight, Hereford, UK) with autoclaved Aspen bedding (Datesand Ltd, UK) and nesting material (‘Sizzle Nest’, Datesand Ltd, UK). Environmental enrichment was provided in the form of chew blocks and cardboard tubes (Datesand Ltd, UK). A seven-day acclimation period was given prior to the start of the study. The animal room was maintained at 23 °C ± 1 °C, 50% ± 10% humidity and on a 12/12 h light dark cycle (lights on at 07:00). Food (CRM(P), SDS Ltd., Essex UK) and tap water were provided ad libitum. Cages were cleaned weekly, ensuring cleaning was not carried out on the day prior to surgery or the day of surgery. Some bedding from the dirty cage was always transferred to the new clean cage. The animals were free from any common pathogens in accordance with the FELASA health monitoring recommendations. Animals were maintained as specific pathogen free according to the FELASA Guidelines (Mähler et al., 2014) and sentinel mice were screened at least quarterly by diagnostic specialists using FELASA approved heath monitoring reports.

### 2.3 Baseline recordings

One week prior to surgery, mice were filmed twice, consecutively, each time in a slightly different set up to allow A) collection of close up HD images of their faces and B) HD footage of the behaviour of the individual mouse within a standard size home cage. A) Mice were placed individually into small custom made chambers (80 × 80 × 80 mm) and close up, high definition (HD) images of their faces recorded during a 3-min session a high definition camera (Casio EX-ZR100, Casio Computer Co., Ltd., Japan). B) Mice were then immediately placed individually in clear plastic cages (350 × 200 × 140 mm) (Techniplast UK Ltd,UK) that contained only sawdust bedding (DBM Ltd, UK). The behaviour of each individual was recorded, in high definition (HD), for 10 min using a video camera (Sony High Definition HandyCam model HDR-XR155, Sony, Japan) positioned at a fixed distance from the cage. The fixed distance was to ensure the whole cage remained in screen shot at the maximum possible resolution throughout. Following filming the mice were returned to their home cages.

### 2.4 Surgery

Thirty minutes prior to the individual’s surgery start time, mice were weighed and administered 0.05 mg/kg buprenorphine subcutaneously (Vetergesic’, Reckitt-Coleman, Hull, UK). This dose and route were chosen based upon recommendations of Dobromylskyj et al. (2000), Robinson et al. (2003), Flecknell (2009). Surgery began at 09:00 h, with the same surgeon operating on all mice. Anaesthesia was induced in a perspex anaesthetic induction chamber (VetTech Solutions Ltd, Cheshire, UK) with isoflurane in oxygen (induction 5%, 2 L/min) for approximately 2 min. The mice were then placed on bedding (VetBed, Kennel Needs and Feeds, Morpeth, UK) on a heating blanket (Harvard Apparatus, Edenbridge, Kent, UK) to maintain body temperature and anaesthesia was maintained using a facemask at 2.5%, 0.5 L/min. The lower abdomen was shaved and the skin sprayed with chlorhexidine (Hydrex Derma spray, Adams Healthcare, Leeds, UK) when loss of the pedal reflex was confirmed. Surgery involved a 1 cm transverse incision made through the skin and abdominal wall. The testes were exteriorized, vas deferentia were located and a small piece removed using cautery, the testes were then returned to the abdomen. The incision in the abdominal wall was closed with Vicryl 5.0 (Johnson & Johnson, Belgium). Tissue glue (Nexaband, Abbott Laboratories, Chicago, IL, USA) and sutures (Vicryl 5.0) were used to close the skin. Anaesthesia lasted 10 ± 2 min, following which the mice recovered in an incubator maintained at 30 °C for 30 min. They were then transferred to a quiet room for filming. No intraoperative complications were reported and all mice recovered from anaesthesia uneventfully.

### 2.5 Post surgery filming

Thirty minutes, five hours and twenty-four hours following the individual’s surgery time, the process of recording footage for facial images (A) followed by recording footage of mouse behaviour (B) was repeated under identical conditions (see Baseline recordings). Following the 24-h post surgery filming, all mice were administered 5 mg/kg meloxicam (s.c.). One-hour post injection, images of the face and video recordings were repeated to determine if this further dose of analgesia was of benefit to the mice.

### 2.6 Data collection

A 6-min clip from each mouse at each time point was manually scored using continuous sampling according to the ethogram shown in Table 1 with ‘Cowlog 2.11’ behavioural software (Hänninen and Pastell, 2009). Analysis was limited to only 6 min since previous research has shown this to be a sufficient time frame to determine if differences are present in behaviour following surgery (Wright-Williams, 2007). Minutes 3–9 of the clip were analysed in order to allow the mouse a short acclimatisation period to the cage. Following manual analysis, the frequency and duration data of each behaviour observed were exported to Microsoft Excel.

Images of the face were taken as screen shots from the high definition video recordings. An image was taken on every occasion a clear view of the mouse’s face was visible with the exception of when the mouse was grooming. These images were then cropped, leaving only the face of the mouse in shot and to prevent bias due to body posture (Langford et al., 2010). Using a random number generator (www.random.org), one image per mouse, per time point was selected. Using the random number generator, the selected images were re-ordered and inserted into a custom designed excel file for scoring. Two observers who were blinded to the experimental details, design and purpose scored every photograph for the five facial action units comprising the MGS as described by Langford et al. (2010). The MGS manual was provided to the scorers for reference, but the title of the manual was edited to ‘mouse facial action coding manual’ to limit bias of scores from the title. Each observer was asked to familiarise themselves with each action unit before commencing scoring of the images. This method has already been shown to lead to high inter-observer reliability and accuracy (e.g. Langford et al., 2010). The MGS score given by each observer, for each mouse, at each time point was calculated, then a mean score produced.

### 2.7 Statistical analysis

Data were analysed using SPSS software (Version 21, IBM). Behaviour data and MGS data were analysed non-parametrically. A Friedman’s test was used to compare MGS and behaviour scores over time. Significant differences between time points were compared using a Wilcoxon test with an adjusted Bonferroni correction for multiple comparisons being applied where appropriate. Results were considered statistically significant when p < 0.05.

## 3 Results

### 3.1 Mouse grimace scale

**Fig. 1:**
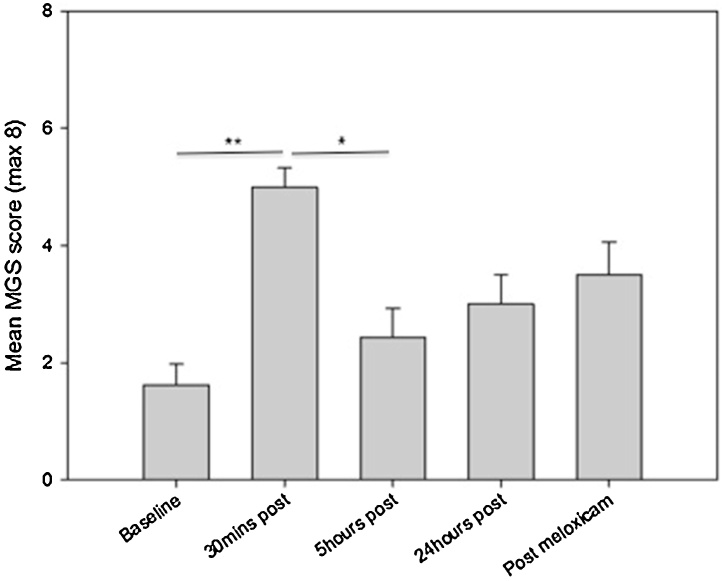
Mean MGS score (±SEM) at each time point. The whisker FAU was excluded from the analysis (maximum score obtainable was 8). *p < 0.05, **p < 0.01.

Due to the high number of failed attempts to score the whiskers, this FAU was excluded from analysis. Maximum MGS score was therefore eight. This issue has been seen in other MGS studies (e.g. Leach et al., 2012). MGS scores were compared across the five time points (Baseline, 30 min, 5 h, 24 h and 25 h). At 30 min post vasectomy, the mean MGS score was significantly greater than at baseline (p < 0.05, z = −2.52, r = −0.89) and at 5 h post vasectomy (p < 0.05, z = −2.53, r = −0.89) (Fig. 1). There were no other significant differences found between the time points.

### 3.2 Behaviour

**Fig. 2:**
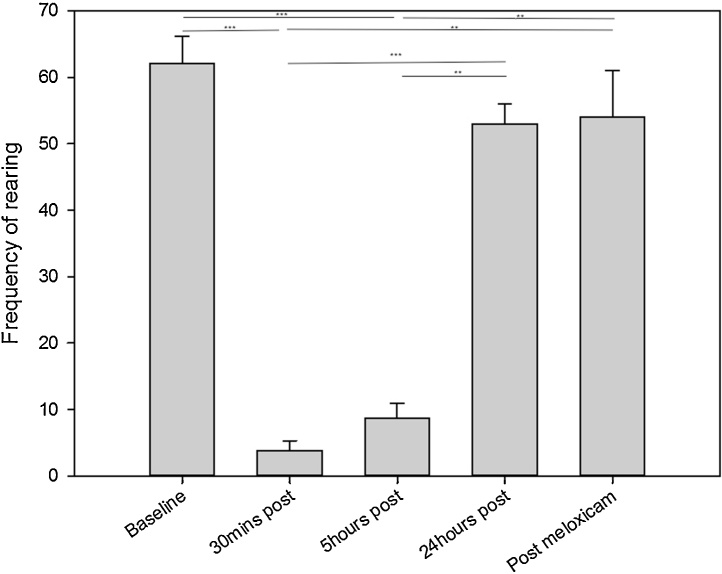
The mean frequency of rearing (±SEM) at each time point, during a 6-min observation period ** p < 0.01, ***P < 0.001.

**Fig. 3:**
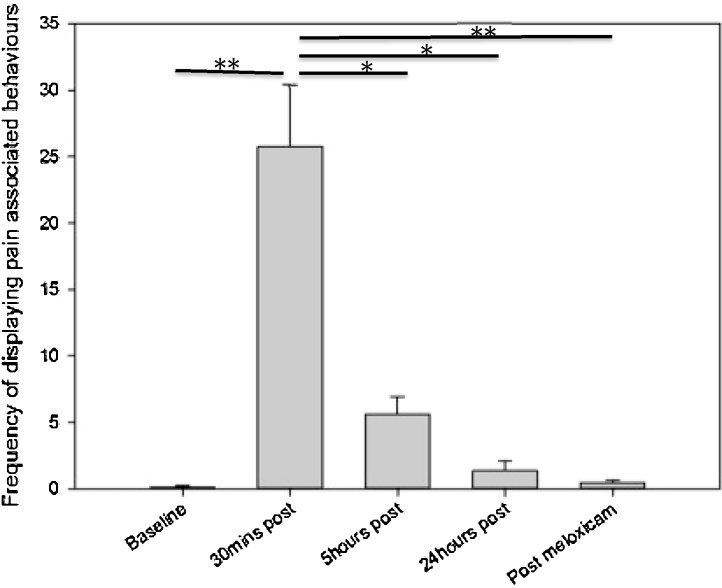
The mean frequency of pain behaviours (stagger, belly press, flinch, rear leg lift, twitch and writhe) (±SEM) at each time point, during a 6-min observation period. * p < 0.05, **p < 0.01.

The frequency of composite pain behaviour (stagger, belly press, flinch, rear leg lift, twitch and writhe) and rearing, and the duration of grooming were compared over the five time points (Baseline, 30 min, 5 h, 24 h and 25 h). There was no significant difference in the duration spent grooming between the five time points (p = 0.42). The frequency of rearing was significantly greater at baseline compared to 30 min post vasectomy and 5 h post vasectomy (p <0.001, z = −2.52, r = −0.89 in both comparisons). The frequency of rearing was significantly greater at 24 h post vasectomy and following a dose of meloxicam than at 30 min post surgery (p < 0.001, z = −2.52, r = −0.89 and p < 0.01 z = −2.37, r = −0.84 respectively) and at 5 h post surgery (p < 0.001, z = −2.52, r = −0.89 and p < 0.01 z = −2.37, r = −0.84 respectively) (Fig. 2). The frequency of pain behaviours was significantly greater at 30 min post vasectomy compared to baseline, 5 h post, 24 h post and following a dose of meloxicam (p < 0.01, z = −2.52, r = −0.89; p < 0.05, z = −2.52, r = −0.89; p < 0.05, z = −2.52, r = −0.89 and p < 0.01, z = −2.37, r = −0.84 respectively) (Fig. 3). There were no other significant differences found between the time points.

## 4 Discussion

Mice undergo surgical procedures as a necessary part of various research protocols. Analgesic efficacy varies between different strains of mice (Wright-Williams et al., 2013) and hence use of “standard” dose rates of agents may not be effective in some strains. Rapid “cage-side” methods of assessing pain and hence analgesic efficacy are therefore needed. Behavioural assessment of pain is highly time consuming (Wright-Williams, 2007), and so detailed manual analysis to identify changes in behaviour that are specific to each strain of mouse limit the number of analgesic regimens that can be studied. Previous studies have identified some key behaviours in mice associated with vasectomy, including belly pressing, twitching and writhing. A composite score of these key pain associated behaviours is useful in analysis of drug efficacy as they individually occur extremely rarely in control animals and are found to significantly reduce in number in painful animals that have received an analgesic (Wright-Williams et al., 2007, Leach et al., 2012, Miller et al., 2015). Here, we studied a different strain of mice, CBA, and scored these specific pain behaviours associated with this procedure. Additionally, we assessed the mice using the MGS to determine if this new, rapid method of pain assessment could be useful in CBA mice following vasectomy.

All mice were administered 0.05 mg/kg buprenorphine pre-emptivley based upon recommendations by Dobromylskyj et al. (2000), Robinson et al. (2003) and Flecknell (2009). Isoflurane is widely used in the laboratory for anaesthetising mice for surgical procedures. Previous work has demonstrated that neither isoflurane or 0.05 mg/kg buprenorphine had a significant effect on MGS score, pain associated behaviours or rearing in pain-free male CBA mice (Miller et al., 2015). Here, the significant increase in pain associated behaviours and MGS score displayed following surgery suggests that buprenorphine at this dose (0.05 mg/kg) was not effective in CBA mice undergoing abdominal approach vasectomy, at a time when the analgesic effect (30mins post-surgery) should be at its greatest based on data from nociceptive tests (Gades et al., 2000). The inability to completely ameliorate pain using this dose has also been demonstrated in other commonly used strains of mice e.g. C3H and C57Bl/6 (Wright-Williams et al., 2013), although a significant reduction in the presence of pain associated behaviours was seen. The significant reduction of pain behaviours and MGS score by 5 h post surgery and return to baseline by 24 h suggests that the most critical time period for the provision of analgesia to mice is in the first few hours immediately post surgery. Mice are very driven to explore their surroundings (Agiriga et al., 2011) and rearing is a key behaviour carried out while exploring a new environment, such as an odour free filming cage containing only clean bedding. Therefore, the significant lack of rearing at 5 h post-surgery is a key indicator of compromised welfare as it represents a reduction in exploration highlighting this period of time as the most critical to monitor mice following vasectomy.

Here, we provided an additional dose of meloxicam at 24 h post surgery and further studied the behaviours and facial expressions of the mice to determine if any benefit could be demonstrated. The key behaviours and MGS that we assessed had returned to baseline levels by 24 h post surgery and were not altered by the administration of meloxicam, indicating that this additional dose of analgesia was unlikely to be of further beneficial at this time point.

The MGS score did increase in line with the presence of validated pain associated behaviours, indicating it may be a suitable method of assessment in CBA mice in the immediate post operative period supporting findings in other mouse strains (e.g. Langford et al., 2010, Leach et al., 2012). Frequency of rearing should also be considered, as this behaviour showed longer lasting deviations from baseline, suggesting a slightly longer compromise in welfare than the pain behaviours and MGS.

Previously validated pain behaviour e.g. twitching and writhing occur very infrequently in baseline scenarios. It is therefore often incorrectly assumed baseline MGS scores are also zero. As strain variation in baseline MGS strains is apparent (Miller and Leach, 2015), using a within subjects design for MGS analysis would be of benefit, rather than solely considering the absolute value scored.

In order for the MGS to be practically useful in a clinical scenario, live scoring of the mice would need to be carried out, rather than retrospectively from still images. Previous study has shown that when scored live, MGS scores are significantly lower than when scored retrospectively from still images (Miller and Leach, 2015). Live vs. retrospective MGS scoring in CBA mice has not yet been directly compared. This would therefore need to be fully investigated prior to replacing time consuming behavioural analysis with this method.

## 5 Conclusion

Pain associated with abdominal vasectomy in CBA does not appear to be ameliorated by the administration of 0.05 mg/kg buprenorphine. The pain induced by the surgery appears to have reduced by 24 h post-surgery. The additional routine administration of 5 mg/kg of meloxicam at 24 post-surgery did not alter any of the pain indices measured indicating that an additional dose of meloxicam is unlikely to have been beneficial. Using these measures alternative analgesics or a higher dose should be investigated to provide more effective pain relief for this model immediately post surgery, with repeat dosing if required.

## Conflicts of interest

The authors declare that they have no competing interests.

## **Author**’**s** contributions

Conception and design of study: AM PF ML, Acquisition of data: AM BS GK ML, Analysis and interpretation of data: AM GK ML, Drafting manuscript: AM PF ML, Final approval of manuscript: AM GK BS PF ML.

## Figures and Tables

**Table 1:** Ethogram used when scoring behaviour in CBA mice pre and post vasectomy. * Indicates behaviours that were categorised as ‘pain behaviours’.

Behaviour	Definition
Belly press*	Pressing of abdomen toward cage floor
Flinch*	Small movement involving whole body
Raised tail*	When walking, tail is lifted from
Rear Leg Lift*	Lifting one rear leg straight out behind
Stagger*	Partial loss of balance when walking
Twitch*	Rapid contraction of back muscles
Writhe*	Contortion of abdominal muscles
Full rear	Standing on rear legs
Partial rear	Standing on rear legs to half stretch
Grooming	Grooming of head, face, back, abdomen, limbs or tail
